# Neutrophil depletion reduces edema formation and tissue loss following traumatic brain injury in mice

**DOI:** 10.1186/1742-2094-9-17

**Published:** 2012-01-23

**Authors:** Ellinor Kenne, Anna Erlandsson, Lennart Lindbom, Lars Hillered, Fredrik Clausen

**Affiliations:** 1 Department of Physiology and Pharmacology, Karolinska Institutet, Stockholm, Sweden; 2 Department of Neuroscience, Neurosurgery, Uppsala University, Uppsala, Sweden

**Keywords:** Neutrophil, traumatic brain injury, brain edema, controlled cortical impact, neuroprotection, blood-brain-barrier, cell death, microglia, neutrophil-depletion, mouse.

## Abstract

**Background:**

Brain edema as a result of secondary injury following traumatic brain injury (TBI) is a major clinical concern. Neutrophils are known to cause increased vascular permeability leading to edema formation in peripheral tissue, but their role in the pathology following TBI remains unclear.

**Methods:**

In this study we used controlled cortical impact (CCI) as a model for TBI and investigated the role of neutrophils in the response to injury. The outcome of mice that were depleted of neutrophils using an anti-Gr-1 antibody was compared to that in mice with intact neutrophil count. The effect of neutrophil depletion on blood-brain barrier function was assessed by Evan's blue dye extravasation, and analysis of brain water content was used as a measurement of brain edema formation (24 and 48 hours after CCI). Lesion volume was measured 7 and 14 days after CCI. Immunohistochemistry was used to assess cell death, using a marker for cleaved caspase-3 at 24 hours after injury, and microglial/macrophage activation 7 days after CCI. Data were analyzed using Mann-Whitney test for non-parametric data.

**Results:**

Neutrophil depletion did not significantly affect Evan's blue extravasation at any time-point after CCI. However, neutrophil-depleted mice exhibited a decreased water content both at 24 and 48 hours after CCI indicating reduced edema formation. Furthermore, brain tissue loss was attenuated in neutropenic mice at 7 and 14 days after injury. Additionally, these mice had a significantly reduced number of activated microglia/macrophages 7 days after CCI, and of cleaved caspase-3 positive cells 24 h after injury.

**Conclusion:**

Our results suggest that neutrophils are involved in the edema formation, but not the extravasation of large proteins, as well as contributing to cell death and tissue loss following TBI in mice.

## Background

The pathological response following traumatic brain injury (TBI) consists of the primary and secondary injury. The primary injury results in death of neurons and glial cells and widespread axonal damage at the moment of impact or acceleration/deceleration. This primary injury initiates a complex secondary injury cascade that includes intracranial inflammation and edema formation. Due to the non-expandable skull compartment, brain edema leads to increased intracranial pressure which in turn causes reduced perfusion and oxygenation of the tissue [1]. After a few days or weeks, the secondary injury cascade has left TBI patients with a much larger brain lesion and contributed to mortality among patients who survived the initial trauma [2].

Recruitment of polymorphonuclear leukocytes (PMN), specifically neutrophil granulocytes, is characteristic of the early inflammatory response following human TBI [3]. Neutrophil recruitment has been shown to increase over the first 24 hours after experimental TBI [4-6], and is dependent on both leukocyte CD11/CD18 [7] and endothelial intercellular adhesion molecule-1 (ICAM-1) [8]. It is well documented that neutrophils trigger alterations in vascular permeability leading to plasma leakage and edema formation in acute inflammation in peripheral tissue [9,10]. More specifically, this relies on neutrophil adhesion to the endothelial lining via β_2_-integrins since functional blockade of the integrin adhesion molecule CD11/CD18 abolishes not only recruitment but also PMN-dependent plasma leakage [11]. The involvement of neutrophils in brain edema formation following TBI remains obscure and evidence exists both to support [5,12], and refute their contribution to blood-brain barrier (BBB) disruption [4,13,14].

When neutrophils are recruited to a site of injury or infection they release a plethora of mediators such as reactive oxygen species (ROS), proteases and pro-inflammatory cytokines, all of which have the potential to adversely affect the integrity of the BBB [15]. The release of elastase and matrix metalloproteases from neutrophils has been shown to increase edema formation in animal models of stroke [16,17]. In accordance, depletion of neutrophils was found to result in an attenuated leakage of proteins across BBB following stroke [18]. Furthermore, cerebral PMN accumulation was correlated with increased intracranial pressure and brain water content after cryogenic brain injury [19].

Although several studies have suggested an important role for neutrophils in edema formation in the central nervous system (CNS) following stroke [20], the present study aims at clarifying the disputed role of neutrophils following TBI. Using the controlled cortical impact (CCI) model, which results in an ipsilateral cortical contusion and cavitation as well as scattered neuronal loss in the underlying hippocampus [21,22], we investigated edema formation in mice with normal levels of neutrophils and in mice that were depleted of neutrophils. In addition, the effects of induced neutropenia on brain tissue loss, apoptosis and microglia/macrophage activation after TBI were evaluated.

## Methods

### Animals and treatment

Male C57Bl/6 mice (Scanbur, Stockholm, Sweden) were kept at 24°C, with 12 h light-dark cycles, and food and water *ad libitum*. Experiments were approved by the regional ethical committee for animal experimentation (reference number: C66/9) and followed the rules and regulations of the Swedish Agricultural Board. Neutrophil depletion (PMN depl) was achieved by i.p. injection of anti-Gr1 mAb RB6-8C5 (100 μg, BioXCell, West Lebanon, USA) 12 hours prior to injury and again 12 hours after [23]. The antibody was administered i.p. to obtain a sustained depletion over the first 48 hours of the experiment. Differential white blood cell count using Türk staining in a Bürker chamber was performed at the time of injury to confirm that the PMN depletion was successful. The experiments were set up according to Table 1. The animals experienced a small weight loss the day after TBI, but started to gain weight day 3 after injury and had recovered to original weight at day 7. There was no significant difference between the treatment groups.

**Table 1:** Experimental set up.

Sample size				Time of sacrifice	Analysis
		
Naïve	Naïve + PMN depletion	CCI	CCI + PMN depletion		
		8	5	24 hours	Evan's blue extraction (EB injection given 4 hours after CCI)

		7	7	24 hours	Cleaved caspase-3 Neutrophil accumulation

6	6	8	8	24 hours	Brain water content

2	3	5	6	48 hours	Evan's blue extraction (EB injection given 24 hours after CCI)

		9	9	48 hours	Brain water content

		5	5	7 days	Microglial/macrophage activation Brain tissue loss

		5	5	14 days	Brain tissue loss

### Controlled Cortical Impact (CCI)

CCI is one of the most widely used and characterized models of TBI in rodents [21,24]. Anesthesia was induced with inhalation of 4% isoflurane in air. During surgery, general anesthesia was maintained with a mix of isoflurane (1.4%) and N_2_O/O_2 _(70/30%), delivered through a nose cone. Mice were placed in a stereotaxic frame and core temperature was maintained at 37°C using a heating pad controlled by a rectal thermometer. Local anesthesia (Marcain, AstraZeneca, Sweden) was applied to the scalp and the skull was exposed by an incision along the midline. A craniotomy (4 mm diameter) was made over the right parietal cortex between the sutures of bregma and lambda using a dental drill. The cortical contusion was delivered by a 2.5 mm diameter piston set to an impact depth of 0.5 mm from a pneumatically driven CCI device (VCU Biomedical Engineering Facility, Richmond, VA, USA). The velocity of the piston was set to 2.8 m/s. The bone fragment was put back in place, secured with tissue adhesive (Histoacryl, Braun, Germany), and the scalp sutured. Naive mice did not undergo any surgical intervention or anesthesia. Animals were sacrificed at indicated time points (Table 1) with an overdose of pentobarbital (300 mg/kg, Apoteket, Sweden).

### Brain water content

Immediately following sacrifice with pentobarbital, the brain was divided along the midline and the contralateral and ipsilateral tissue was weighed immediately following removal to obtain wet weight (WW). The tissue was then dried at 60°C for 72 hours and weighed to obtain dry weight (DW). Water content was calculated as a percentage of wet weight; % water content = (WW-DW)/WW × 100.

### Evans blue dye extravasation

Mice were injected with 100 μl Evans blue (EB, 2% in PBS, Sigma) through the tail vein at indicated times (Table 1). Evans blue dye injected intravenously binds instantaneously to albumin and other plasma proteins and serves as a marker for plasma exudation. Animals were sacrificed as described above and perfused with heparinized saline. Brain tissue from the contra- and ipsilateral side was analyzed. The tissue samples were snap frozen in -55°C isopentane and freeze-dried. Freeze-dried specimens were homogenized in formamide (1:20 w/v) and incubated at 60°C overnight. The homogenate was centrifuged at 14000 rpm for 30 min and the EB content in the supernatant was determined spectrophotometrically at 620 nm (Titertek Multiscan).

### Lesion and Hemispheric Volumes

Mice that were sacrificed one (n = 5+5) or two (n = 5+5) weeks after injury were transcardially perfused with heparinized isotonic saline (1000 U/l) and then with phosphate-buffered 4% formaldehyde (Histolab AB, Gothenburg, Sweden). Following rapid removal the brain was placed in 4% formaldehyde in PBS at 4°C for 24 hours and 30% w/v sucrose at 4°C for 72 hours. It was then snap frozen in -55°C isopentane. Seven sections from bregma levels -1 to -4 mm, 500 μm apart, were stained with Mayer's Hematoxylin and Eosin (Histolab) and, using a digital camera (mcm5c; Zeiss Gmbh), photographed in a stereomicroscope (Zeiss Stemi 2000-C; Zeiss Gmbh). The hemispherical volume and cortical lesion volume were calculated using ImageJ (NIH, Bethesda, MD, USA.). Volumes (*n*) were calculated using the formula: Σ_n = 1_^7 ^(*A_n _+ A_n + 1_*) × *d*/2, where *A *is the hemispherical or cortical lesion area and *d *the distance between sections [25]. Tissue loss of the ipsilateral (injured) hemisphere was calculated as a percentage of the contralateral (uninjured) hemispheric volume.

### Immunohistochemistry

**Figure 1:**
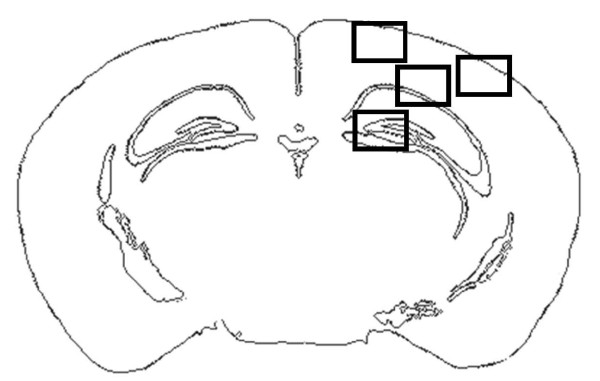
**Three regions (600 × 800 μm) in the cortex and one in the dentate gyrus of the hippocampus were selected for counting of apoptotic cells and activated microglia/macrophages**. The arrow indicates the presence of the contusion.

Immunohistochemistry was used to determine parenchymal cell apoptosis (cleaved caspase-3, n = 7+7) and neutrophil infiltration (Gr-1, n = 7+7) 24 hours after CCI, and microglial/macrophage activation (Mac-2 expression, n = 5+5) 7 days after CCI. Brains were fixed as described above and cryosectioned to 12 μm thick coronal sections, thawed and fixed in acetone for 1 min. Normal horse serum (10%) in PBS with 0.1% Triton-X-100 was used to block unspecific binding. Four sections, from different bregma levels were incubated with antibodies to cleaved caspase-3 (Cell Signaling Technology), Gr-1 (Abcam, Cambridge, U.K.) or Mac-2 (Cedarlane Laboratories, Burlington, ON, Canada) in PBS with 0.3% Triton X-100 (1:200) overnight. Following washing with PBS for 3 × 5 min, sections were incubated with an AlexaFluor-conjugated rabbit-anti-rat secondary antibody (Molecular Probes, Eugene, OR, USA, 1:200) in PBS with 0.1% Triton-X-100 for 30 min. Slides were washed with PBS and mounted using Vectashield with DAPI (Vector laboratories, Burlingame, CA, USA) as a nuclear marker. A fluorescence microscope system (Zeiss Axiovision, Zeiss Gmbh, Göttingen, Germany) was used to capture immunohistochemical images of cleaved caspase-3, Gr-1 and Mac-2 staining at 100× magnification. Images from bregma levels -1.5, -2.0, -2.5 and -3.0 mm were evaluated using Axiovision image analysis software. Three regions of interest (600 μm × 800 μm) in the cortex and one in the dentate gyrus of the hippocampus were evaluated bilaterally in each animal (Figure 1).

### Statistics

Statistical evaluations were made using Statistica (StatSoft, Tulsa, OK, USA). After testing the data for normality, the non-parametric Mann-Whitney test was used. Results are presented as means ± SEM.

## Results

### Controlled cortical impact causes PMN accumulation in cortical tissue

**Figure 2:**
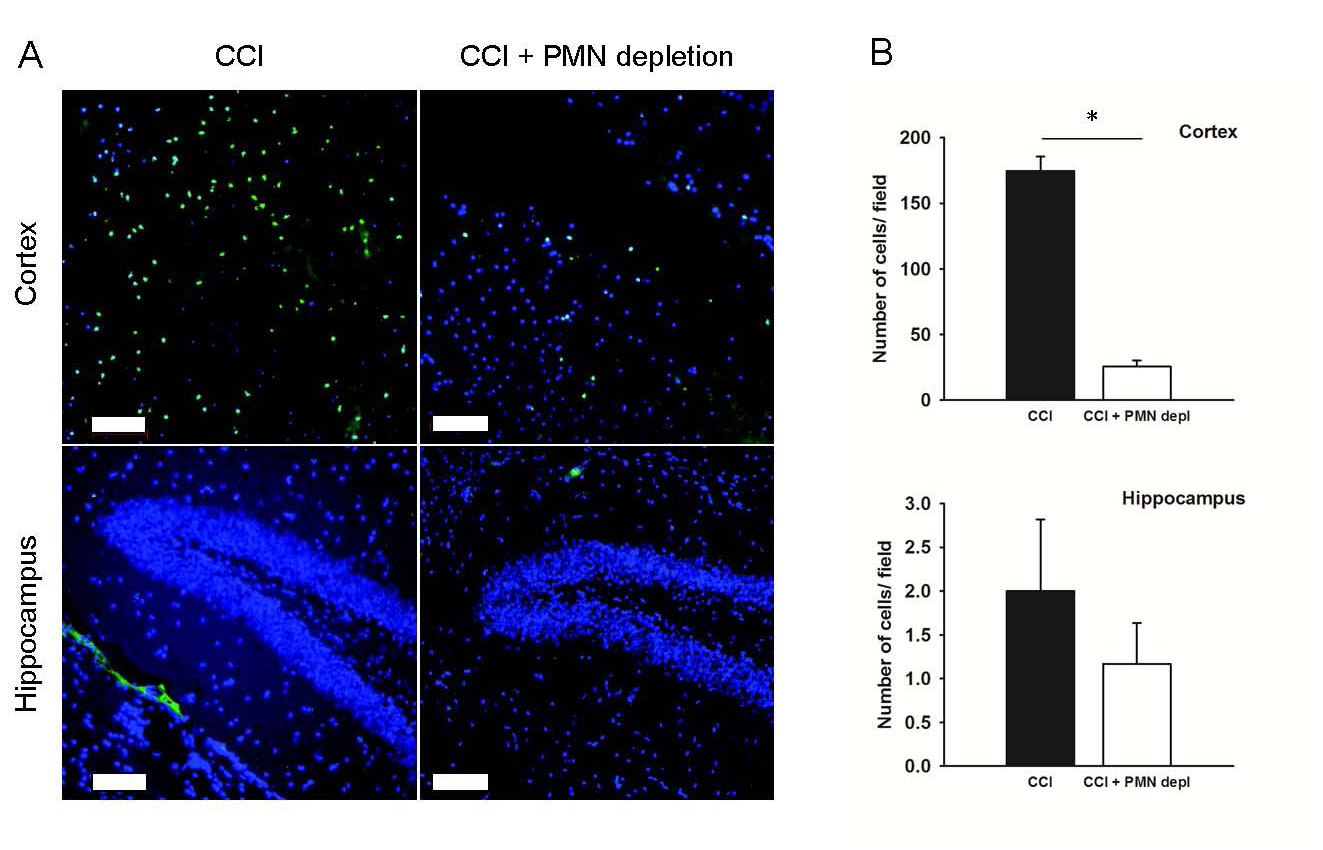
**Neutrophil accumulation in the injured cortex 24 hours after injury is dramatically reduced in neutropenic mice**. A) Micrographs of Gr-1 positive cells (green) 24 hours after CCI in the cortex and hippocampus. Blue represents nuclear staining using DAPI. Scalebar is 100 μm. B) Quantification of Gr-1 positive cells in the cortex and hippocampus of intact (black bars, n = 7) and PMN-depleted (open bars, n = 7) mice. * indicates significant difference at p < 0.05. Values are mean ± SEM.

Controlled cortical impact (CCI) was used as a model for TBI to investigate the role of neutrophils in the injury response. To confirm that the injury resulted in recruitment of PMN, brain sections taken 24 hours after injury were stained with an antibody against the Gr-1 antigen. In line with previous research [3-6], TBI resulted in accumulation of PMN in the injured cortex (174.7 ± 10.7 cells/ field, Figure 2). As expected, this response was significantly reduced (p < 0.05) in the neutropenic animals (25.5 ± 4.7 cells/ field). In contrast to what was observed in the cortex, there was no obvious neutrophil recruitment to the hippocampus (1.2 ± 0.47 cells/ field and 2 ± 0.82 cells/ field for neutropenic mice and control animals, respectively). In the contralateral cortex only a few Gr-1 positive cells were found (2.3 ± 0.09 cells/ field for neutropenic mice and 3.2 ± 0.24 cells/ field for control animals).

### Brain edema following TBI is neutrophil-dependent

**Figure 3:**
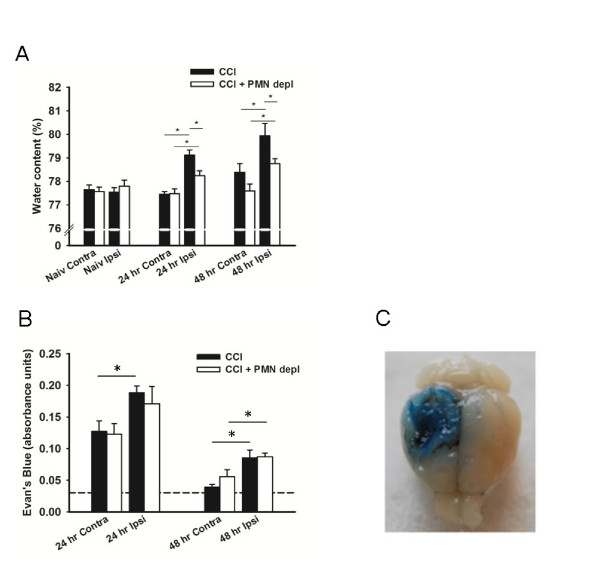
**Brain water content but not macromolecule extravasation following TBI is affected by neutrophil depletion**. A) Brain water content in the contralateral and ipsilateral areas 24 and 48 hours after CCI in intact (black bars, 24 hrs: n = 8, 48 hrs: n = 9) and PMN-depleted (open bars, 24 hrs: n = 8, 48 hrs: n = 9) mice. Values for naïve mice are provided for comparison. B) Evan's blue content in the contralateral and ipsilateral sides 24 and 48 hours after CCI in intact (black bars, 24 hrs: n = 8, 48 hrs: n = 5) and PMN-depleted (open bars, 24 hrs: n = 5, 48 hrs: n = 6) mice. Dashed line represents baseline EB leakage in naïve mice 24 hours after injury for comparison. * indicates significant difference at p < 0.05. Values are mean ± SEM. C) Representative image of EB leakage 24 hours following CCI.

We used two methods to determine edema formation 24 and 48 hours following TBI. These time points were chosen as brain edema following CCI peaks during the first two days [26]. In addition, PMN accumulation following CCI is known to take place 24 and 48 hours after injury [6,22,27]. First, water content in the injured brain was used as a measure of edema after CCI. As expected, water content was significantly higher (p < 0.05) in the ipsilateral side of the brain in mice that received TBI compared to naive animals (Figure 3A). Neutrophil depletion resulted in significantly decreased (p < 0.05) water content in the ipsilateral hemisphere compared to untreated TBI animals both at 24 h (78.2% ± 0.21% vs. 79.1% ± 0.22%) and 48 h (78.8% ± 0.21% vs. 79.9% ± 0.53%) after injury. No statistical difference was observed in the contralateral hemisphere.

To further examine the effects of neutrophil depletion on plasma exudation we used Evan's blue dye as a marker for albumin extravasation (Figure 3C). Baseline leakage of EB was determined in naive mice and there was no difference between neutropenic and intact mice (0.0325 AU ± 0.0013 AU vs. 0.0308 AU ± 0.0030 AU). CCI resulted in a significant (p < 0.05) increased EB extravasation in the injured area compared to the contralateral side or to naive animals at either time point after injury (Figure 3B). However, depletion of PMN did not result in attenuated levels of extracted EB compared to control mice at 24 hours (0.1706 AU ± 0.0269 AU vs. 0.1875 AU ± 0.0107 AU), or 48 hours (0.0325 AU ± 0.0013 AU vs. 0.0308 AU ± 0.0003 AU). Taken together, these data indicate that PMN depletion counteracts the increase in brain water content, but does not protect from BBB breakdown following CCI.

### Neutrophil depletion attenuates tissue loss following TBI

**Figure 4:**
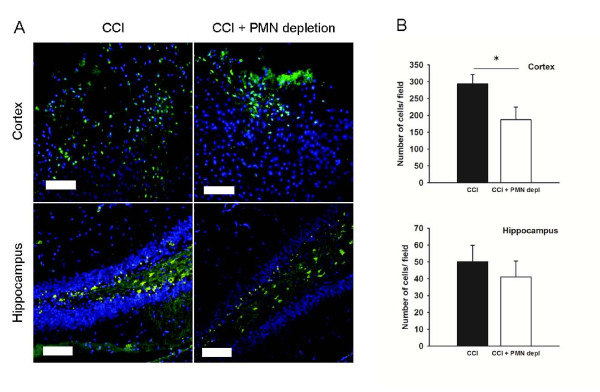
**Tissue loss following TBI is attenuated in neutrophil-depleted mice**. Top panels represent the lesion volume (A) and hemispheric tissue loss (B) one and two weeks after TBI in mice with intact PMN (black bars, n = 5) and mice that were rendered neutropenic (open bars, n = 5). The bottom panel (C) displays representative images of Mayer's Hematoxylin and Eosin stained brain sections that were used for analyses. * indicates significant difference at p < 0.05. Values are mean ± SEM.

To determine the impact of PMN depletion during CCI on injury size later in the disease process, we analyzed lesion volume (Figure 4 A and 4B) and tissue loss, in the injured hemisphere in comparison to the uninjured hemisphere (Figure 4 C and 4D. Induced neutropenia resulted in significantly reduced (p < 0.05) lesion volume two weeks after injury (3.626 ± 0.22 mm^3 ^for control and 2.488 ± 0.23 mm^3 ^for PMN depleted mice). There was a tendency for an attenuated lesion volume also at one week after injury in the PMN depleted mice, although the difference was not significant (3.646 ± 0.58 mm^3 ^for control and 2.208 ± 0.08 mm^3 ^for PMN depleted mice) Furthermore, in neutrophil-depleted mice there was a significant reduction (p < 0.05) of ipsilateral hemispheric tissue loss compared to injured control mice both at seven (5.9 ± 0.85% vs. 10.3 ± 1.61%) and fourteen (10.9 ± 1.1% vs. 17.2 ± 1.7%) days after injury (Figure 4 A and 4B). These data strongly suggests that neutrophil depletion at the time of injury protects from brain tissue damage.

### Neutrophil depletion reduces apoptosis 24 h after injury

**Figure 5:**
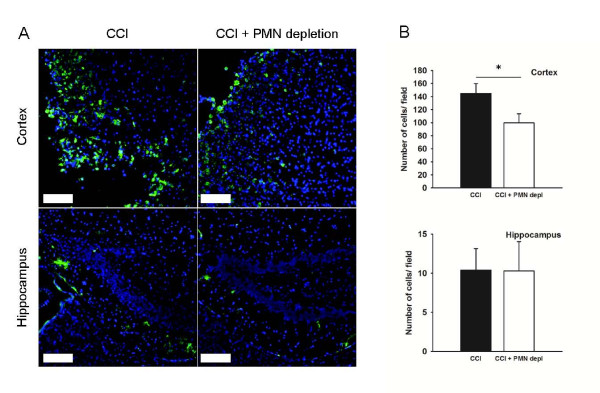
**Neutrophil depletion attenuates the number of cleaved caspase-3 positive cells**. A) Microphotographs of cleaved caspase-3 positive cells (green) 24 hours after CCI in the cortex and hippocampus. DAPI was used for nuclear staining (blue). Scalebar is 100 μm. B) Quantification of cleaved caspase-3 positive cells in cortex and hippocampus of intact (black bars, n = 7) and PMN-depleted (open bars, n = 7) mice. * indicates significant difference at p < 0.05. Values are mean ± SEM.

Apoptotic cells, as determined by cleaved caspase-3 staining, were assessed in the cortex and the dentate gyrus of the hippocampus 24 hours after TBI (Figure 5). As expected, the hemisphere ipsilateral to the injury showed an increased number of apoptotic cells, both in cortex and hippocampus, compared to the contralateral side. Neutropenia significantly reduced (p < 0.05) the number of cleaved caspase-3 positive cells in the cortex (187.3 ± 37.4 cells/ field vs. 293.2 ± 28 cells/ field), but not in the dentate gyrus (41.1 ± 9.5 cells/ field vs. 50.2 ± 9.7 cells/ field), indicating that the accumulation of PMN in the cortex contributes to caspase-3 activation.

### Microglial/macrophage activation is attenuated in neutrophil-depleted mice

**Figure 6:**
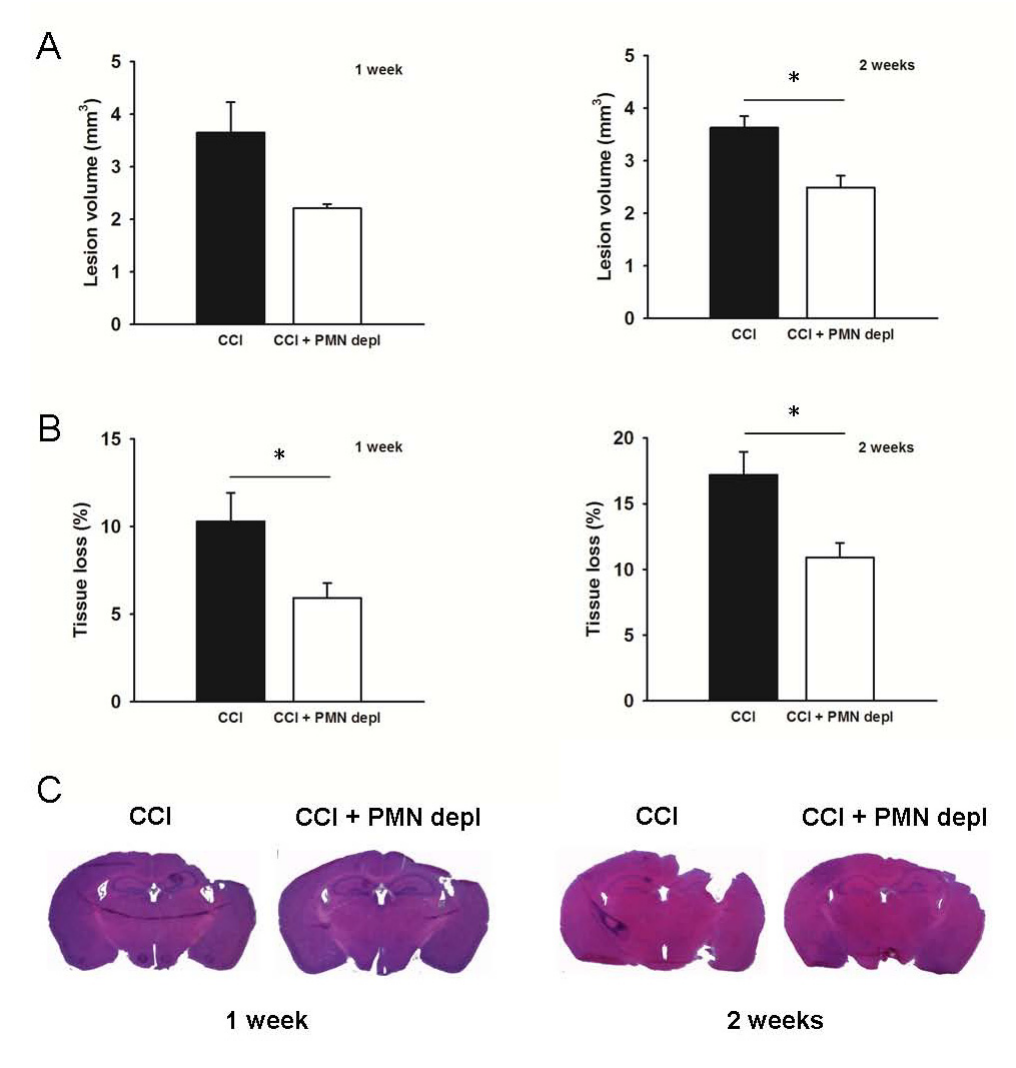
**Microglial/macrophage activation is reduced in neutropenic mice**. A) Microphotographs of Mac-2 positive cells (green) 7 days after CCI in the cortex and hippocampus. DAPI was used for nuclear staining (blue). Scalebar is 100 μm. B) Quantification of Mac-2 positive cells in cortex and hippocampus following CCI (black bars, n = 5) or CCI and neutrophil depletion (black bars, n = 5). * indicates significant difference at p < 0.05. Values are mean ± SEM.

In order to investigate the effect of neutropenia on microglial/macrophage activation, immunostaining with specific antibodies to Mac-2 was performed on brain sections from animals 7 days post injury, based on previous time course studies [22]. The hilus of the dentate gyrus was chosen to evaluate the injury to the hippocampus, as in our hands this area shows the most change after CCI. Mac-2 positive cells in the ipsilateral hippocampus and cortex were assessed using a fluorescence microscope (Figure 6A). There was a large number of activated microglia/macrophages in the cortex after TBI, which was significantly (p < 0.05) reduced in the PMN-depleted group (99.7 ± 14 cells/ field vs. 144.8 ± 15 cells/ field; Figure 6B). On the other hand, counting the number of positive cells revealed that there was only a small number of activated microglia/macrophages in the dentate gyrus of the hippocampus (Figure 6B), 10.3 ± 3.2 cells/ field for neutropenic mice and 10.5 ± 2.2 cells/ field for control animals. These data are in line with a previous study of neutrophil depletion in a model of intracerebral hemorrhage (ICH) [18].

## Discussion

Cerebral edema and secondary injury are feared complications of TBI. Recruitment of neutrophil granulocytes is known to cause increased vascular permeability and edema formation in peripheral tissue. However, the connection in the CNS between neutrophil emigration and edema formation is not clear. Therefore, this study used antibody-induced neutrophil depletion to investigate the role of neutrophils in brain edema formation following CCI in mice. As additional endpoint parameters, we looked at the brain tissue loss, microglia/macrophage activation and apoptosis of cells in the injured area.

Cerebral edema is a consequence of structural and functional changes of the BBB, the microcirculation or of parenchymal cell volume regulation, and can be classified as cytotoxic or vasogenic. Cytotoxic edema occurs as a result of intracellular swelling of glia and neurons and may arise independently of the integrity of the BBB, as a result of energy metabolic crisis and perturbation of ion homeostasis. A disruption of the BBB or disturbances in the microcirculation on the other hand results in vasogenic edema [1]. Increased brain water content is an important clinical feature of TBI potentially resulting in raised intracranial pressure, lowered cerebral perfusion pressure and eventually reduced cerebral blood flow with impaired glucose and oxygen delivery to the brain tissue [1]. Brain water content has been shown to increase following TBI in rodents and remain elevated for up to 7 days [28]. As PMN do not seem to mediate the early BBB breakdown, during the first 4 hours, after trauma [29], we investigated the effects of PMN depletion on cerebral edema formation 24 and 48 hours after injury. By measuring brain water content we show that the edema after CCI is attenuated in mice that are depleted of neutrophils. This attenuation was maintained for at least 48 hours after injury. In order to assess whether disruption of the BBB and plasma extravasation (vasogenic edema) contributed to the increased brain water content during the first days after injury, we used Evan's blue as a marker for macromolecule extravasation. However, our results suggest that the enhanced macromolecular leakage across the BBB 24 or 48 h after injury occurred independently of neutrophils. It is known that neutrophil-adhesion to postcapillary venules in peripheral tissue results in decreased endothelial barrier function and extravasation of macromolecules [10]. Our results indicate that the BBB might respond differently to neutrophil adhesion. This difference could be explained by the morphology, biochemistry and function of the BBB that are distinct from that of the endothelial lining in peripheral tissue [30]. Another possibility is that the mechanical trauma from the CCI results in a breakdown of the BBB that is independent of PMN recruitment. Thus, the strong mechanical impact on the blood vessels and the hemorrhage formed will mask any PMN-dependent leakage that is evident in response to a more diffuse injury [5,12,31]. Moreover, cerebral edema and macromolecular leakage have been shown not to be temporally correlated [1] indicating that BBB breakdown is not the only factor leading to edema following TBI. For example, osmotic brain edema caused by imbalances between blood and brain tissue, e.g. hyponatremia, is a common feature of clinical TBI [1]. It is therefore possible that the edema formation may increase without EB leakage being affected [32], perhaps explaining the results of the present study.

Possible mechanisms behind the neutrophil-dependent tissue swelling in our study could be release of ROS and proteases such as matrix metalloproteinases (MMPs) in the parenchymal tissue leading to breakdown of cells and a cytotoxic edema. Modulation of free radicals seems to improve several parameters, such as edema formation, injury size and neurological score, after TBI [33]. PMN derived substances such as MMPs and ROS, have been shown to have a direct cytotoxic effect on neuronal cells *in vitro *[34], which could explain the increasing brain water content seen after injury. The cytotoxic effects of PMN may explain the attenuation in apoptotic cell count in neutropenic animals. However, it is difficult to discern whether the apoptotic cells cause the edema or vice versa.

Acute injury to the brain activates the microglia and the release of pro-inflammatory cytokines [35]. It has been shown that neutrophils may stimulate recruitment and activation of monocytes/macrophages in peripheral tissue [36]. Here, we show that microglial/macrophage activation is less prominent in mice that are rendered neutropenic, which suggests a similar relationship between neutrophil recruitment and phagocytic cell activation in the brain as in peripheral tissue. This relationship is further strengthened by the lack of PMN accumulation in hippocampus, which is associated with few apoptotic cells and activated microglia/macrophages. Our findings are supported by previous research using a model of intracerebral hemorrhage in rat, where neutropenia resulted in significantly reduced number of activated microglia/ macrophages 7 and 14 days after injury [18]. The results described in this study might also be due to an attenuation of the secondary injury caused by PMN thus requiring less activation of microglia/macrophages.

In addition to decreased apoptosis, we show that neutrophil depletion results in attenuated brain tissue loss and lesion volume following TBI. The neuroprotective effect of neutropenia was significant at both 7 and 14 days after injury. Thus, there is a beneficial effect of early PMN depletion in the injury development, possibly due to less PMN-related cytotoxicity, less edema or a reduced number of inflammatory cells in the injured area. Several studies have shown an association between attenuated edema formation and reduced tissue loss following experimental TBI [24,37,38]. However, the role for PMN has not been elucidated. It has previously been shown that blocking ICAM-1 results in improved neurological scores following brain injury, possibly due to reduced PMN recruitment [39]. In addition, decreased neutrophil recruitment as a result of a deficiency in the chemokine receptor CXCR2 correlated with reduced tissue damage following closed head injury in mice [40]. Further, an attenuation of the acute inflammatory response and edema formation was associated with decreased neuronal damage and behavioral deficits 28 days after TBI [41]. Moreover, when comparing two different models of TBI (weight drop and CCI), it was shown that the weight drop model gave an increased PMN accumulation, which was associated with a larger lesion volume in those animals [27], providing additional support for the role of PMN in tissue loss.

PMN may aggravate cerebral injury by several mechanisms; especially their ability to secrete MMPs, ROS and cytokines have been implicated in this respect [34]. Inhibiting any of these factors were shown to be neuroprotective in *in vivo *models of TBI [33,42-44], making the assumption that PMN activation and infiltration is involved in the secondary injury after TBI highly plausible. ROS scavenger treatment lowered ICAM-1 expression and reduced neutrophil recruitment to the rat brain following TBI resulting in attenuated morphological brain damage [6]. In addition, inhibiting the recruitment of PMN to the brain following ischemia-reperfusion injury prevents the increase in MMP-9 [45]. Data like these may explain why treatments resulting in decreased PMN recruitment could lead to attenuated infarct size following ischemia-reperfusion injury [46,47]. Our results further strengthen the role for PMN in the tissue damage following TBI.

## Conclusion

In this study we show that neutrophils have a role in edema formation following TBI, possibly most influential on the cytotoxic edema. Furthermore, early neutrophil depletion is effective in reducing the tissue loss that arises secondary to the injury. This association between PMN-induced brain edema and neuronal damage is a novel link in the disease progress following TBI and interference with neutrophil recruitment may be a complementary treatment in the management of TBI.

## List of abbreviations

BBB: blood brain barrier; CCI: controlled cortical impact; DW: dry weight; EB: Evan's blue; ICAM-1: intercellular adhesion molecule-1; ICH: intracerebral hemorrhage; MMPs: matrix metalloproteases; PMN: polymorphonuclear leukocytes; PMN depl: neutrophil depletion; ROS: reactive oxygen species; TBI: traumatic brain injury; WW: wet weight.

## Competing interests

The authors declare that they have no competing interests.

## Authors' contributions

EK and FC performed experiments, analyzed data, and contributed to writing of the manuscript. AE, LH and LL participated in the design and coordination of the study as well as helped to draft the manuscript. All authors read and approved the final manuscript.
